# Canine visceral leishmaniasis in an urban setting of Southeastern Brazil: an ecological study involving spatial analysis

**DOI:** 10.1186/s13071-014-0485-7

**Published:** 2014-10-20

**Authors:** Rafael Gonçalves Teixeira-Neto, Eduardo Sérgio da Silva, Renata Aparecida Nascimento, Vinícius Silva Belo, Cláudia di Lorenzo de Oliveira, Letícia Cavalari Pinheiro, Célia Maria Ferreira Gontijo

**Affiliations:** Centro de Pesquisas René Rachou, FIOCRUZ, Avenida Augusto de Lima 1715, Barro Preto, 30190-002 Belo Horizonte, MG Brazil; Universidade Federal de São João del Rei, Campus Dona Lindu, Av. Sebastião Gonçalves Coelho 400, Chanadour, Chanadour, 35501-296 Divinópolis, MG Brazil; Escola Nacional de Saúde Pública, FIOCRUZ, Rua Leopoldo Bulhões 1480, Manguinhos, 21041-210 Rio de Janeiro, RJ Brazil; Universidade Federal de Juiz de Fora, Campus Governador Valadares, Rua Israel Pinheiro 2000, 35020-220 Governador Valadares, MG Brazil

**Keywords:** Visceral leishmaniasis, Epidemiology, Spatial analysis, Statistical tools, Disease control

## Abstract

**Background:**

The physical characteristics of the environment influence the composition, distribution and behavior of the vectors and mammalian hosts involved in the transmission of visceral leishmaniasis (VL), thereby affecting the epidemiology of the disease. In Brazil, urbanization of human VL is a recent phenomenon and represents an issue of particular concern to local health authorities. The present study aimed to establish the degree of spatial dependency between canine and human VL in the municipality of Divinópolis, Minas Gerais, Brazil, and to identify priority risk areas in which stricter control measures should be implemented.

**Methods:**

The selected canine population comprised 3,652 dogs distributed within 11 strata and 1,247 urban blocks. Serum samples were collected between March 2013 and February 2014. Serodiagnosis of dogs was performed using the enzyme-linked immunosorbent assay and the indirect fluorescent-antibody test. The blocks sampled for canine VL and the addresses of the 16 confirmed cases of human VL notified in Divinópolis during the period 2007–2013 were georeferenced. Spatial analysis of the data was performed using Kernel density estimation, Ripley’s bivariate K-function and directional distribution methods.

**Results:**

The overall prevalence of seropositive animals was 4.63% (range 3.95 - 5.31) (*n* =169) and varied in different strata between 0.9 (range 0.0 - 1.91) and 8.73% (range 5.65 - 11.81). A positive spatial dependency was detected between human and canine VL in which the occurrence of human cases of the disease tended to concentrate in locations that were close to areas with a higher incidence of canine VL. The priority risk area could be clearly distinguished from Kernel density estimation and standard deviational ellipse plots in which the human VL ellipse was totally enclosed within the canine VL ellipse.

**Conclusions:**

The results presented herein will enable the Municipal Health Office of Divinópolis to devise a more effective management plan for human VL in which specific strategies would be applied to areas presenting different levels of risk. This spatial evaluation of leishmaniasis model could be applied in other urban areas of Brazil.

## Background

Visceral leishmaniasis (VL) is a severe infectious disease that can result in death if not diagnosed and treated in a timely manner. It is estimated that, on a worldwide basis, more than 500,000 new cases of VL occur every year resulting in 51,000 VL-related deaths [1,2]. Although the disease is endemic in 87 countries, 66 of which are located in Africa, Asia and Europe and 21 in the Americas, the vast majority (~90%) of notified cases occur in Bangladesh, Brazil, Ethiopia, India, South Sudan, and Sudan [3].

Historically, VL was characterized in Brazil as a rural endemy [4], but during the early 1980s the disease suffered an epidemiological transformation by spreading to urban areas of the country. Moreover, reports of the occurrence of VL in large urban centers have became increasingly more frequent as shown by studies conducted in the southeastern metropolitan areas of São Paulo [5], Rio de Janeiro [6] and Belo Horizonte [7,8], as well as in the northeastern capitals of Terezina [9], São Luis [10,11] and Fortaleza [12].

The factors responsible for the urbanization of VL in Brazil have received considerable attention, particularly those relating to environmental changes promoted by the rural exodus, the lack of planning and sanitation in urban areas, the adaptation of the main insect vector to urban settings, and the presence of domestic reservoirs of the disease [13-16]. Considering such factors, it is clear that the success of disease control programs will depend on the development of tools that will assist in defining strategies for epidemiological surveillance that target local realities and in facilitating the implementation of appropriate actions.

In 1984, the Brazilian Ministry of Health created a Program for the Monitoring and Control of Visceral Leishmaniasis (*Programa de Vigilância e Controle da Leishmaniose Visceral;* PVCLV), the objectives of which were to diminish the level of morbidity and the rate of mortality associated with VL and to reduce the risks of disease transmission. Management strategies were aimed at the diagnosis and treatment of human cases of VL as early as possible, the control of the vector population (i.e. phlebotomine sandflies), and the elimination of domestic canine reservoirs [17]. Unfortunately, these strategies have not led to a reduction in the number of cases in endemic areas nor have they impeded the emergence of VL at focal points in disease-free areas [18].

Spatial analysis in health is a field of science concerned with understanding the geographical patterns of morbidity and mortality relating to an infectious disease, and the association of these patterns with the characteristics or risk factors responsible for its dissemination. The technique has been widely employed in the study of leishmaniasis and has permitted detailed analyses of the spatial dependency between canine and human VL, the distribution of the vector, and the characterization of areas with high incidence and high risk of morbidity [7,18-27].

The aim of the present study was to perform a spatial analysis of canine VL in the municipality of Divinópolis, state of Minas Gerais, Brazil, in order to establish the spatial dependency between the occurrence of canine and human VL and to identify priority areas for implementing stricter surveillance and control actions.

## Methods

Details of the project were approved by the Ethical Committee in Research of the Universidade Federal de São João Del Rey (UFSJ; protocol number 35/2010), and all procedures were conducted in accordance with the guidelines of the Colégio Brasileiro de Experimentação Animal (COBEA).

### Study site

**Figure 1:**
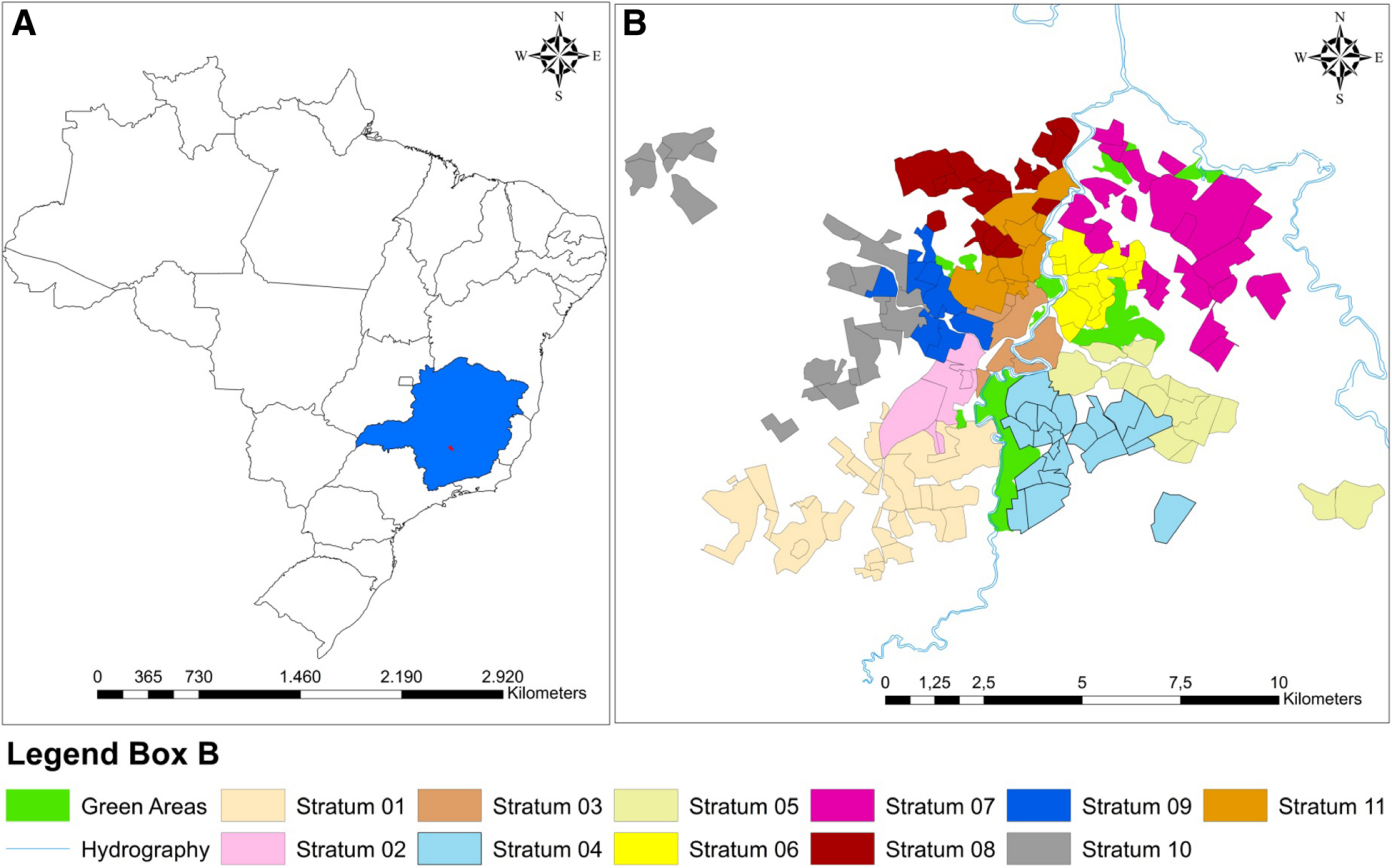
**Maps of Brazil, the state of Minas Gerais (Blue) and the municipality of Divinópolis (Red) (A); Districts of the municipality of Divinópolis (B).** Map **B** shows the districts and principal green areas of the municipality of Divinópolis together with the 11 strata representing the sectors organized according to the plan proposed by the Brazilian Ministry of Health for the eradication of *Aedes aegypti*. Each stratum is divided into districts and blocks, and sampling of dogs was performed in 1,247 blocks.

Divinópolis (20.13889 S; 44.88389 W) is located in the Vale do Itapecerica (state of Minas Gerais) and is home to one of the main centers of the metallurgical industry in the region (Figure 1). The population comprises some 216,000 inhabitants [28]. According to the Municipal Health Surveillance Service, Divinópolis is classified as endemic for cutaneous leishmaniasis and 46 human cases have been notified between 2007 and 2013. Although the first case of human VL was recorded in the area in 2007, with a further 15 new cases notified between 2008 and 2013, no investigation has been carried out concerning the prevalence of canine VL and its relation to the distribution of human leishmaniasis.

### Dog population evaluated

The criteria for including dogs in the serological survey for LCan were that they were domestic animals with owners and that they had been accustomed to living at home. Stray dogs were not included in the study due to their greater rate of movement and the consequent interference in spatial data analysis.

### Epidemiological survey of canine VL

The survey employed proportional stratified cluster sampling in which the strata were the sectors of the Plan for the Eradication of *Aedes aegypti* (*Plano de Erradicação do Aedes aegypti;* PEAa) and the clusters were the blocks located within each district. We chose to use the stratification system based on PEAa because it is recommended by Brazilian Ministry of Health [17] for efforts to control LV. The use of this method can facilitate consistent sampling among municipalities, however, regions that do not have a stratification system based on PEAa, may apply other stratification methods. Pursuant to the PEAa, the council of Divinópolis has stratified the municipality according to the density of the human population by defining 11 strata each with approximately 17,000 inhabitants (Figure 1). Each stratum is subdivided into districts, and these are further segregated into blocks, delimited by streets, with an approximate size of 10,000 to 15,000 m^2^.

In order to estimate the dog population in the city we used data from the latest rabies vaccination campaign. With this information it was possible to estimate the relationship between human and canine populations. Assuming that the geographical distribution of the population of domestic dogs correlates with the distribution of the human population and to ensure the proportional representation of all districts of Divinópolis, the number of dogs tested in each district was directly proportional to the size of the human population in that district. Thus, if the human population of a given district comprised 10% of the total population of the stratum, the canine sample size of the district was taken as 10% of the total number of dogs in the stratum.

In order to calculate the sample size, a table describing the number of animals to be sampled according to the estimated canine population of the district and the expected prevalence of canine VL, considering a level of significance of 5%, was employed [17]. The prevalence of canine VL was set at 3% on the basis of a preliminary study performed by our research group in one restricted area of the municipality. According to the above, the total sample population required was determined to be 3,652 dogs representing 332 animals for each of the 11 strata. The selection of blocks for canine sampling was determined with the help of a random number table, with the number of blocks drawn being based on the total number of dogs to be sampled in each district while maintaining proportional representation in relation to the stratum. The number of dogs to be sampled in each block was calculated by dividing the total number of dogs in the district by the total number of blocks in the same district. In this manner, proper distribution of the sample was maintained by eliminating the possibility of sampling too many animals in a single block.

### Serodiagnosis of canine VL

Blood samples were collected from animals, transferred to filter papers and stored in the freezer at −20°C until required for analysis. Following the introduction of The Brazilian Ministry of Health Guidelines that were effective until 2013, diagnosis of infection was performed through enzyme-linked immunosorbent assay (ELISA) and confirmation of seropositivity was achieved by indirect fluorescent-antibody test (IFAT) using the respective EIE-LVC and IFI-LVC serological kits (Instituto de Tecnologia em Imunobiológicos, Bio-Manguinhos, Rio de Janeiro, Brazil) according to the instructions provided by the manufacturer. Serum samples were collected between March 2013 and February 2014. The assays were carried out in the Laboratory of Parasitology at UFSJ, which is accredited by the Brazilian Ministry of Health for the performance of such tests.

### Georeferencing

All blocks in which dogs had been sampled, together with the addresses of the VL patients notified during the period 2007–2013, were georeferenced using a Garmin GPSMAP 76S (Olathe, KS, USA) hand-held global positioning unit. We included all human cases of visceral leishmaniasis that had a diagnosis confirmed by a laboratory certified by the Brazilian Ministry of Health and that were reported by the municipal health department between the years 2007 and 2013. The geographic coordinates were recorded at the centers of the blocks, the positions of which were ascertained by reference to Google Earth® (Mountain View, CA, USA). Data were stored in a database created especially for the study and plotted on the map of Divinópolis. Mapping and spatial analyses of the data were performed using ESRI ArcGIS™ version 10.0 (Redlands, CA, USA) and R version 3.0.2 (R Development Core Team, Wirtschaftsuniversität Wien, Vienna, Austria) software with the aim of identifying VL distribution patterns.

### Ripley’s bivariate K-function analysis

The dependency between cases of canine and human VL was determined using Ripley’s K12-cross function, which indicated whether the point patterns within a study area were either independent of one another, or exhibited attraction (the two types of events tended to occur close together), or exhibited repulsion (the two types of events tended to occur far apart) [29]. Ripley’s K12-function was defined as:

$$ K(d)=\frac{1}{\lambda_2}E\left({N}_{2d}\right) $$

where, *E*(*N*_*2d*_) is the expected number of type 2 events within a distance of up to *d* of an arbitrary type 1 event, and *λ*_2_ is the total density of type 2 events in the study area.

Ripley’s K12-functions were plotted using R software version 3.0.2, and envelopes of bivariate functions were constructed from random toroidal shifts in order to detect patterns of spatial association between the two types of point patterns. If the curve is inside the envelope the two point patterns are spatially independent, otherwise they are spatially dependent. In the case of spatial dependency, the relationship may be positive (when the curve is above the upper line of the envelope) or negative (when the curve is below the lower line of the envelope).

### Kernel image segmentation

Kernel density estimation is a clustering technique that has been applied to image segmentation in order to produce hotspot maps showing spatial trend patterns of an infectious disease [30]. In this study, each observation was weighted according to its distance from a central value (nucleus), thereby creating a continuous surface representing the density of VL in which hotspots (clusters) were highlighted. The search radius was fixed at 300 m, and the corresponding density map was plotted using ESRI ArcGIS™ version 10.0 software.

### Directional distribution of human and canine VL

The standard deviational ellipse method was used to summarize the spatial characteristics of VL clusters in terms of central tendency, dispersion and directional trends. Ellipses, which were plotted using ESRI ArcGIS™ version 10.0 software, marked areas with higher concentrations of infection and provided information about the asymmetry and distribution of the data. The long axis of each ellipse defined the direction of maximum dispersion, whereas the short axis (perpendicular to the long axis) revealed the direction of minimum dispersion.

## Results

**Figure 2:**
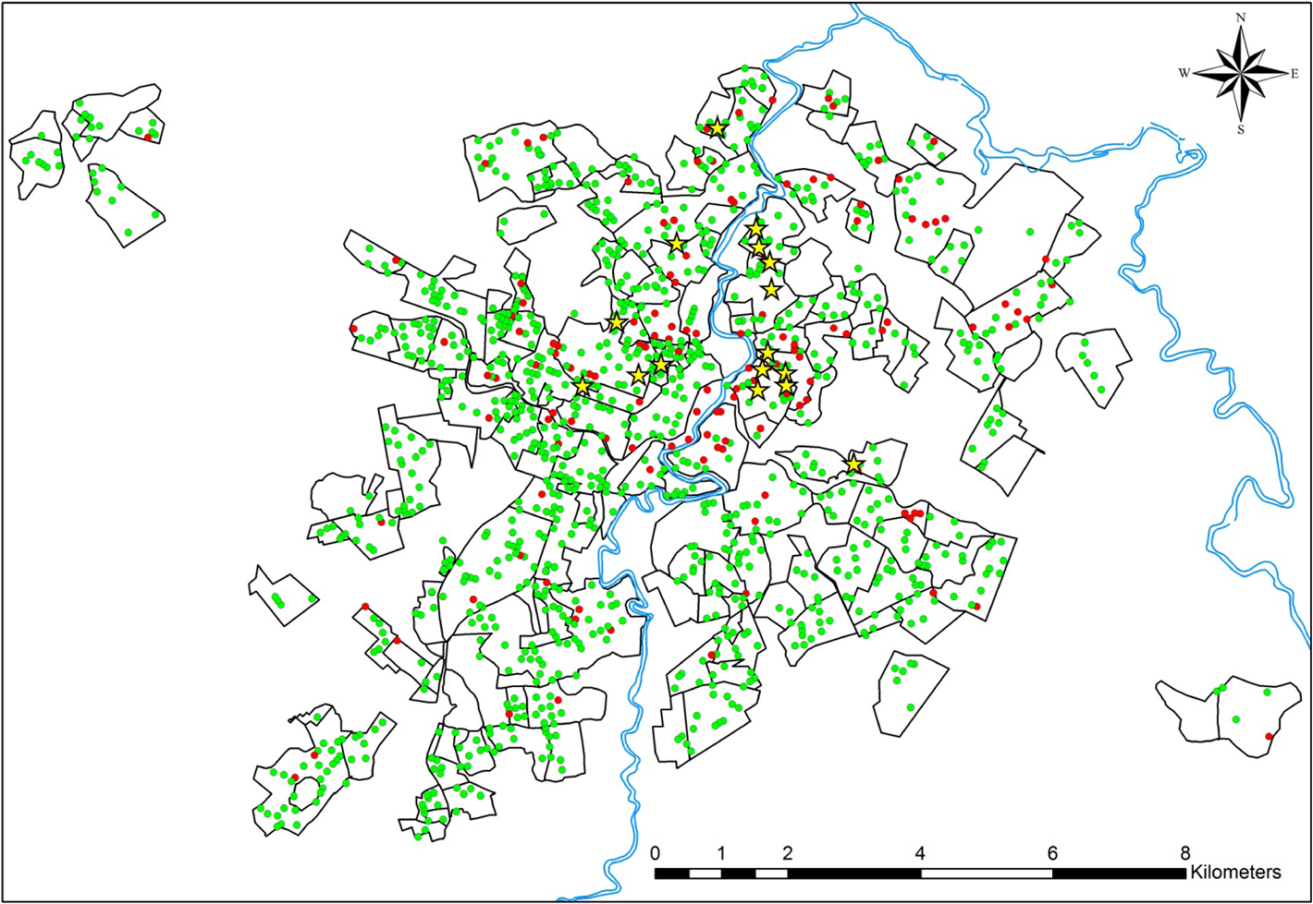
**Districts of the municipality of Divinópolis.** Yellow stars represent the human cases of visceral leishmaniasis recorded during the period 2007–2013, while the red and green dots represent the 1,247 sampled blocks in which the serological survey was performed with presence or absence of infected dogs respectively.

A total of 1,247 blocks in the municipality of Divinópolis were sampled and their geographical coordinates are represented as red and green dots on the map presented in Figure 2. Of the 3,652 dogs surveyed within these blocks, 169 were seropositive for VL presenting an overall prevalence of 4.63% (range 3.95 - 5.31%). The addresses of the 16 confirmed cases of human VL notified during the period 2007–2013 were also georeferenced and are represented as yellow stars on the map shown in Figure 2.

**Figure 3:**
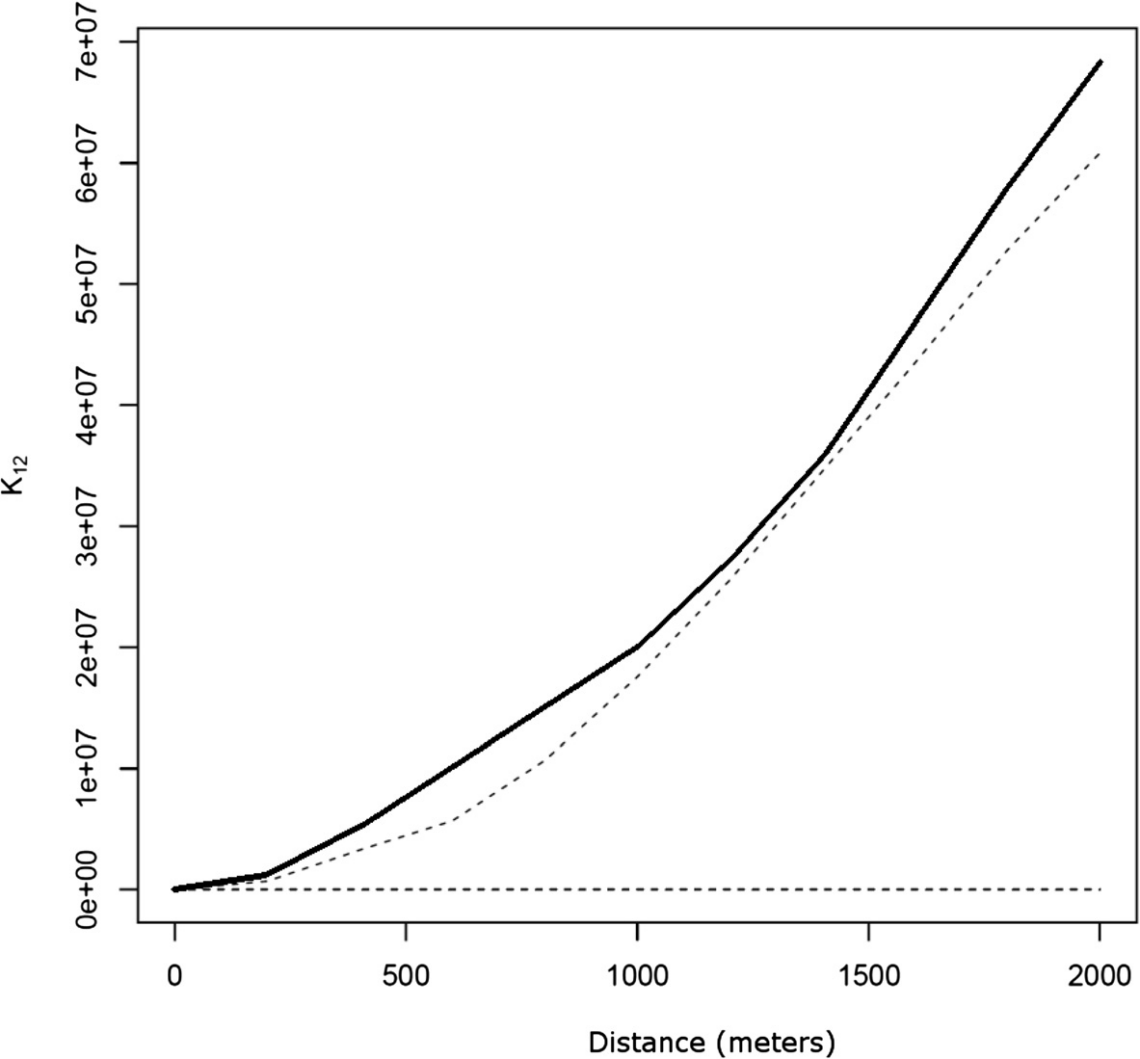
**Ripley’s bivariate K-function analysis.** The black continuous curve above the upper line of the envelope demonstrates the positive spatial dependency between canine and human visceral leishmaniasis in Divinópolis.

**Figure 4:**
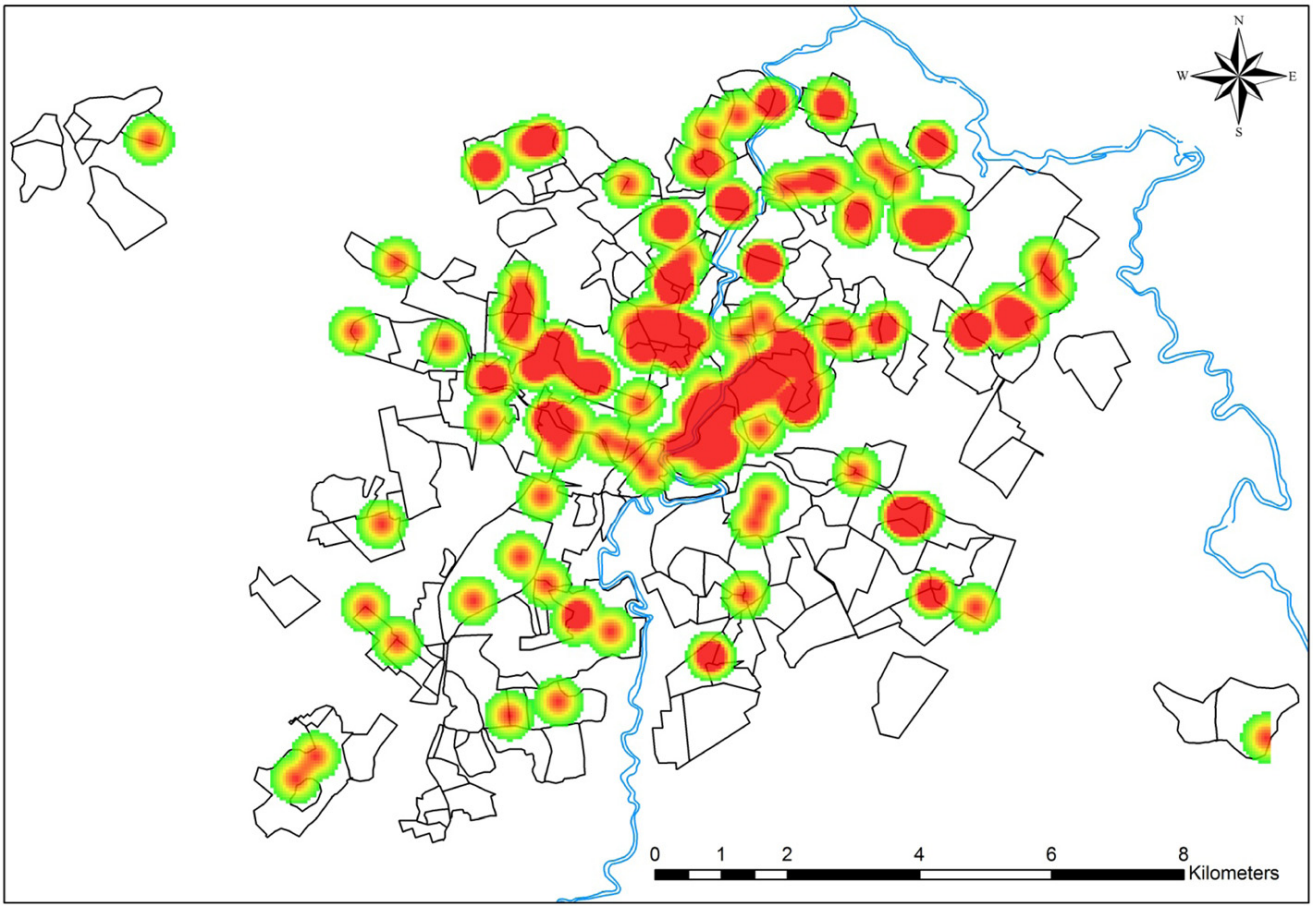
**Kernel density map showing the distribution of canine visceral leishmaniasis in Divinópolis.** The red spots represent the areas where the density of seropositive dogs was more observed.

**Figure 5:**
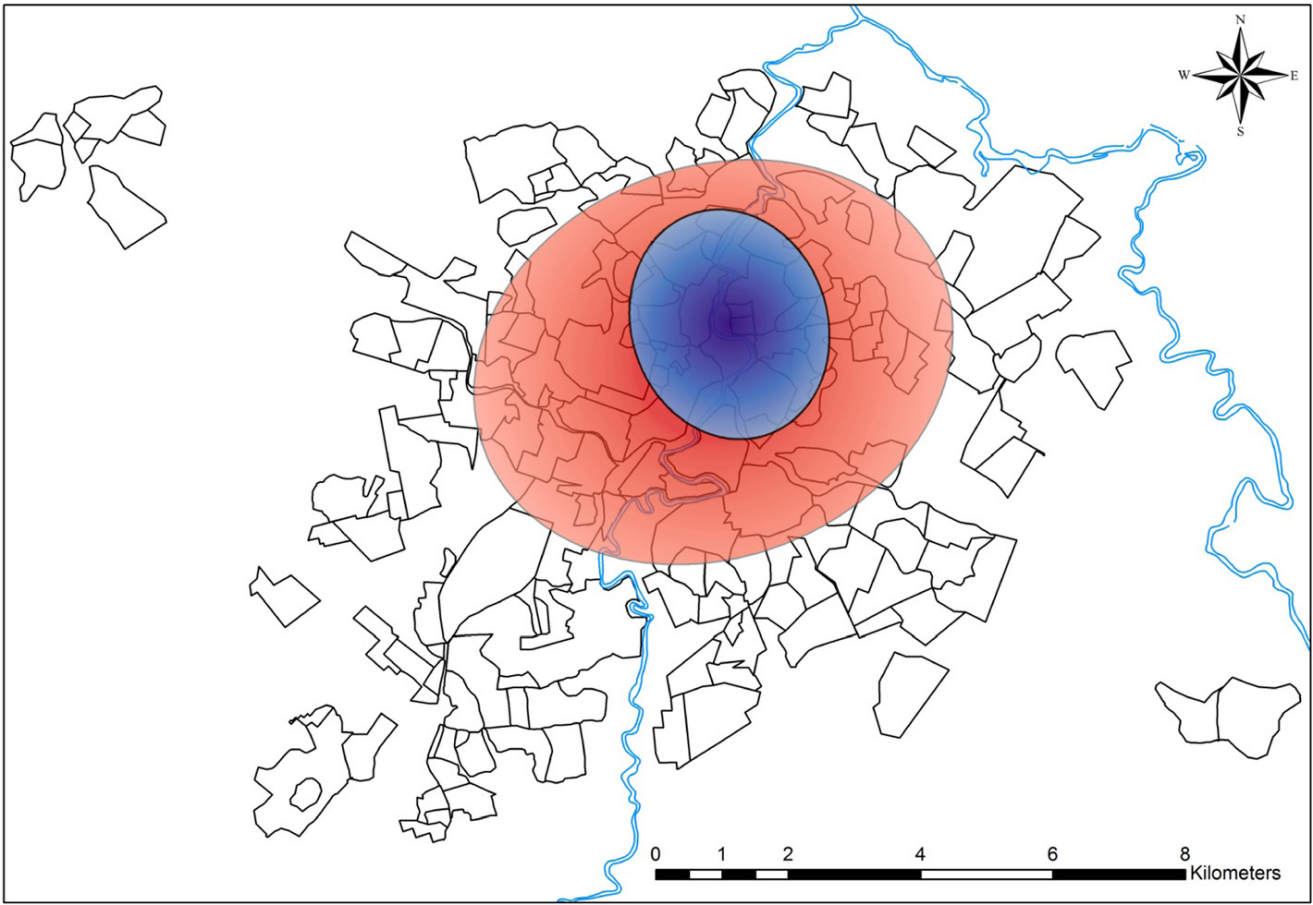
**Directional distribution of canine (red ellipse) and human (blue ellipse) visceral leishmaniasis in Divinópolis.** The 16 cases of human visceral leishmaniasis were notified during the period 2007–2013.

The Ripley’s K12-function plot depicted in Figure 3 indicates a positive spatial dependency between human and canine VL in which the occurrence of human cases of the disease in Divinópolis tended to concentrate in locations that were close to areas with a higher incidence of canine VL. Analysis of the Kernel density map (Figure 4) revealed that canine VL was widely distributed in the municipality with seropositive dogs identified in all 11 strata. However, the prevalence of the disease varied considerably, ranging from 0.9% (range 0.0 - 1.91%) in stratum 2 to 8.73% (range 5.65 - 11.81%) in stratum 7 (Table 1). Priority areas for the implementation of stricter measures to control VL could be clearly identified from the directional distribution of canine cases revealed in the standard deviational ellipse plots of human and canine VL (Figure 5). It is noteworthy that the human VL ellipse was included inside the much larger canine VL ellipse.

**Table 1:** **Distribution and seroprevalence of canine visceral leishmaniasis in Divinópolis, Brazil, between March 2013 to February 2014**

**Stratum** ^**a**^	**Sampled dogs (** ***n*** **)**	**Seropositive dogs (** ***n*** **)**	**Prevalence of canine VL (%)** ^**b**^
01	332	10	3.01 (1.14 - 4.88)
02	332	3	0.90 (0.0 - 1.91)
03	332	16	4.82 (2.48 - 7.16)
04	332	6	1.81 (0.35 - 3.27)
05	332	11	3.31 (1.36 - 5.26)
06	332	28	8.43 (5.4 - 11.46)
07	332	29	8.73 (5.65 - 11.81)
08	332	16	4.82 (2.48 - 7.16)
09	332	18	5.42 (2.95 - 7.89)
10	332	6	1.81 (0.35 - 3.27)
11	332	26	7.83 (4.90 - 10.76)
Total	3,652	169	4.63 (3.95 - 5.31)

^a^Divinópolis is divided into 11 strata representing the sectors organized according to the plan proposed by the Brazilian Ministry of Health for eradication of *Aedes aegypti*. Each stratum is subdivided into districts and each district is subdivided into blocks as represented in Figure 2.

^b^95% confidence interval shown in brackets.

## Discussion

While human VL is a serious public health problem throughout Brazil, the continued spread of the disease within urban areas represents a particular cause for concern [7-12]. Moreover, despite the efforts of the Brazilian health authorities, human VL continues to expand [31], indicating the ineffectiveness of the PVCLV.

The urbanization of VL is not a phenomenon exclusive to Brazil, however, since it has been described in other countries including Iran [32], Mexico [33] and Morocco [34]. A number of factors are believed to be responsible for the urbanization of the disease and these include operational and technical difficulties in eliminating the potential reservoirs, failure in controlling the propagation of the vector, and the high cost of control measures [35]. These failures contribute to the persistence of reservoirs and vectors in urban centers, a situation that not only sustains the disease cycle but also contributes to its expansion into disease-free areas.

In Brazil, epidemiologists are of the opinion that a key issue responsible for the failure of PVCLV is the difficulty of allocating municipal financial resources to the program [36]. Considering this shortfall in funding, it is clearly of fundamental importance to adjust control policies and actions to the specific realities of the areas concerned [8,17].

In Divinópolis, the first cases of canine VL were identified by our research group in 2003, at which time it was possible to isolate and characterize the parasite *Leishmania infantum* in dogs originating from the local society for the protection of animals (unpublished data). Four years elapsed until the first notification of human VL in 2007 by the Municipal Health Surveillance Service of Divinópolis, and this has been followed by 15 additional cases in subsequent years (2008–2013).

Epidemiological studies carried out in various urban areas have concluded that canine VL generally precedes human VL. Moreover, canine VL is considered the primary cause of all registered outbreaks since there are no reports of the occurrence of human VL in the absence of infected dogs [35]. The association between canine and human VL was suggested in studies conducted more than 10 years ago. Thus, Camargo-Neves *et al.* [36] showed that a high incidence of human VL occurred in areas where canine VL was more prevalent, while Oliveira *et al.* [7] reported that the spatial distribution of VL in Belo Horizonte suggested a correlation between the two forms of the disease. More recently, the presence of *Leishmania*-infected dogs was considered a risk factor for human VL in urbanized environments [24]. In addition, some epidemiological studies support the hypothesis that dogs are the main reservoir of *L. infantum* in the urban environment [13,37]. It is of interest to note that the association between human VL and infected dogs has also been reported by researchers from countries such as Iran [38] and Uzbekistan [39].

A determining factor in the dissemination of VL in Divinópolis is the presence of the principal vector of *L. infantum*, namely, the phlebotomine sandfly. More than 17 species of the subfamily Phlebotominae have been collected in the urban area of Divinópolis, demonstrating the existence of higher species richness in comparison with other towns in Minas Gerais endemic for VL. Seven of the 17 phlebotomine species identified in Divinópolis were confirmed or suspected vectors of *Leishmania spp.*, among which *Lutzomyia longipalpis* was the most abundant [40]. The rapid dissemination of VL among the canine population and the emergence of the disease among humans of Divinópolis results from the presence of the agents of disease transmission and *Leishmania*-harboring reservoirs (dogs), and the inability of PVCLV to break the transmission cycle.

Geographic information systems and spatial analysis of infectious diseases have become common tools and are used widely by Brazilian researchers working in the field of leishmaniasis [7,18,19,22,24-27]. Spatial analysis allows the visualization of regions with the highest prevalence of the disease and assists in the identification of associated risk factors and in the design of appropriate management strategies. Moreover, knowledge of the geographical context increases the ability to predict disease patterns and to identify target areas likely to be at the highest risk, thereby reducing the overall costs of control programs. Cutting costs is essential for the success of PVCLV since the program places a heavy financial burden on the municipal public health system [14,41]. Real and Biek [42] have emphasized the need to recognize the spatial heterogeneities that exist in different urban settings since the physical attributes of the environment may modulate the genetic structure and the spatial dynamics of host–pathogen interactions. In the case of VL, there has been much concern about the importance of characterizing different cities with the purpose of proposing specific strategies for low- and high-risk areas. Thus, Costa *et al.* [43] have emphasized the need to identify priority risk areas within an endemic region and to apply control measures according to the prevalence of infected dogs. These authors state that the removal of asymptomatic seropositive animals should be intensified in areas with greater prevalence of canine VL even though the strategy of euthanasia remains somewhat controversial. Following spatial analysis of human and canine VL in São Luis, Barbosa *et al.* [25] were able to establish priority risk areas and suggested that those with a higher density of infected dogs should be preferentially targeted. Araujo *et al.* [24] performed spatial analysis in some districts of Belo Horizonte and identified the areas with the highest risk of VL together with their associated risk factors. According to these authors, the relative risk of human VL was positively correlated with the density of infected dogs.

Some limitations of the study should be mentioned: i) not evaluating stray animals may result in an underestimation of the prevalence of canine infection. According to a recent review, stray animals are at a higher risk of being infected than are domestic animals [37]; ii) we did not use spatial analysis techniques that evaluate the interpolation of data and that provide relevant information about the animals that live in non-sampled regions. Considering that local digital databases have limitations that hinder the application of more sophisticated methods of spatial analysis, we chose to use tools that are easy to implement and interpret, even by professionals not accustomed to statistical spatial analysis.

The spatial distribution of VL may not be stable, so it is important to understand the epidemiological realities of each region in order to develop more effective control measures. In spite of local peculiarities, the presence of infected dogs is considered by many researchers to be one of the major risk factors associated with human VL [13-15] and among the epidemiological variables that favor the spread of the disease the presence of domestic dogs is the most stable. We should clarify that the use of this method for defining risk areas provides an initial assessment of the distribution of the disease in an urban center and it should be followed-up with additional research. Other spatial analysis techniques that evaluate the interpolation of data, as well as studies involving stray animals and the presence of wild and synanthropic vectors and reservoirs, should also be implemented. With the addition of such studies a more realistic epidemiological assessment and documentation of the dispersion of the disease can be made.

Perhaps the techniques applied in this study (Kernel image segmentation, Ripley’s K12 function and directional distribution analysis) were efficient in determining not only the positive dependency between canine and human VL in Divinópolis but also the potential highest priority risk area. This area could be clearly distinguished from standard deviational ellipse plots in which the human VL ellipse was totally enclosed within the canine VL ellipse. However, it is necessary to keep in mind that we mapped only symptomatic human cases, and as such they may not reflect the actual spatial distribution of all human cases of VL. In order to better identify risk areas asymptomatic human cases need to be included as well before a more accurate assessment of the spatial distribution of human VL can be achieved. These techniques can be readily applied to other urban settings.

## Conclusions

The results presented herein can assist the Municipal Health Office (Secretaria Municipal de Saúde) of Divinópolis to devise an appropriate disease management plan whereby specific strategies would be applied to different risk areas. The identification of risk areas based on the distribution of infected domestic dogs may serve as a directional study in that it allows the evaluation of the most efficient way to apply other epidemiological assessment strategies. Studies of the distribution of CanL should be developed continuously and include the spatial distribution of vectors, wild and synanthropic reservoirs, and stray dogs in order to contribute to a more complete understanding of the mechanisms of spatial dispersion of LV in large urban centers.

In conclusion, the PVCLV should use a risk management approach since the control and prevention of VL depends entirely on disrupting the transmission cycle of *L. infantum*. Thus, mapping high and low risk areas in urban settings is crucial to the success of such programs in large urban centers in Brazil.
